# Surplus Killing of Magellanic Penguins *Spheniscus magellanicus* by Puma *Puma concolor* ) in Islote Lobos National Park, Northern Patagonia

**DOI:** 10.1002/ece3.74585

**Published:** 2026-10-27

**Authors:** Jesica D. Hombre, Magdalena Arias, Esteban Tazzioli, Raúl González, Enrique Crespo

**Affiliations:** ^1^ Centro de Investigación Aplicada y Transferencia Tecnológica en Recursos Marinos Almirante Storni (CIMAS, CONICET) San Antonio Oeste Río Negro Argentina; ^2^ Administración de Parques Nacionales, Dirección Regional Patagonia Norte San Carlos de Bariloche Río Negro Argentina; ^3^ Facultad de Ciencias Marinas FaCiMar, U. N. Comahue San Antonio Oeste Río Negro Argentina; ^4^ Centro de Ciencias Naturales, Ambientales y Antropológicas Universidad Maimónides y Fundación Azara Buenos Aires Ciudad Autónoma de Buenos Aires Argentina; ^5^ Centro de Estudios de Sistemas Marinos (CESIMAR, CENPAT, CONICET) Puerto Madryn Chubut Argentina

**Keywords:** land–sea trophic interactions, Magellanic penguin, Patagonia, puma, surplus killing

## Abstract

Surplus killing (SK) by large carnivores may generate high‐intensity mortality events in colonial seabirds. Here, we document repeated predation events involving Magellanic penguins ( *Spheniscus magellanicus* ) and pumas ( *Puma concolor* ) in Islote Lobos National Park (ILNP, Río Negro, Argentina). During the 2024–2025 breeding season, only four adult penguins with cervical injuries and no signs of consumption were recorded, precluding their interpretation as SK. In contrast, during the 2025–2026 breeding season, multiple high‐intensity mortality events were recorded at La Pastosa and Isla de los Pájaros, two islets harboring reproductive penguin colonies in the ILNP. Across all documented 2025–2026 events, at least 961 penguins were found dead, including 887 adults and 74 chicks. Carcasses exhibited cervical bite injuries and little or no evidence of consumption, while fresh puma tracks, feces, and direct observations of an adult individual within the colonies strongly supported puma predation. During the four consecutive breeding seasons prior to these events, population monitoring did not record mortality episodes of comparable magnitude and suggested stable population dynamics within the ILNP colonies. The mortality recorded during the 2025–2026 breeding season represented a minimum direct mortality of 3.52% of the local breeding adult population. These records represent the northernmost confirmed case of SK involving Magellanic penguins and pumas, extending the documented geographic range of this interaction by more than 1000 km northwards. They also suggest that repeated SK events may represent an important source of additive mortality for local penguin colonies. Our findings highlight the ecological importance of episodic terrestrial–marine trophic interactions and the need for continued demographic monitoring in coastal breeding systems.

## Graphical abstract

We document repeated surplus killing events by pumas ( *Puma concolor* ) affecting breeding colonies of Magellanic penguins ( *Spheniscus magellanicus* ) in Islote Lobos National Park, northern Patagonia, Argentina. During the 2025–2026 breeding season, at least 961 penguins were killed, representing the largest natural mortality event recorded in the colony to date. These observations highlight the potential demographic importance of episodic natural mortality events in seabird populations.

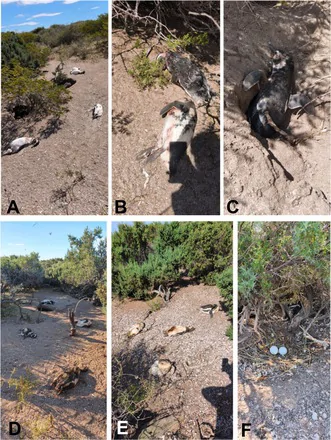

## 1 Introduction

Predation by large carnivores is a natural ecological process, yet under conditions of high prey availability and limited escape opportunities predators may kill more prey than required to meet their immediate energetic needs, a phenomenon known as surplus killing (SK) (Kruuk 1972; Linnell et al. 1999; Lucherini et al. 2018). This behavior has been documented in several carnivore taxa, including canids and felids, and is commonly associated with temporally concentrated prey resources and restricted prey escape capacity (Murphy and Macdonald 2010).

Trophic interactions at the land–sea interface play an important role in ecosystem dynamics, linking marine‐derived resources with terrestrial consumers (Polis and Hurd 1996). In this context, seabird colonies may function as highly concentrated and seasonally predictable prey resources for terrestrial predators, particularly in mainland breeding sites or colonies that become temporarily accessible from the coast (Lera et al. 2026). Because reproduction is constrained to land, seabirds are especially vulnerable to terrestrial predation during the breeding season (Jones 2001).

In Patagonia, Magellanic penguins ( *Spheniscus magellanicus* ) breed in numerous mainland and insular colonies distributed along the Atlantic coast between approximately 40°–55° S (Boersma et al. 2013, García‐Borboroglu et al. 2022, Hombre et al. 2026). Recent studies from southern Patagonia documented repeated puma ( *Puma concolor* ) incursions into mainland colonies of Magellanic penguins at Monte León National Park, including multiple killing events consistent with SK behavior and evidence of recurrent terrestrial–marine trophic interactions (Serota et al. 2023; Lera et al. 2026). These studies suggest that dense penguin colonies may function as episodic prey resources for terrestrial carnivores under favorable access conditions.

Here, we report repeated high‐intensity predation events involving Magellanic penguins and pumas in Islote Lobos National Park (ILNP, Río Negro, Argentina). These records extend the documented northern distribution of this interaction along the Argentine coast and provide new evidence of recurrent predator incursions into breeding colonies of Magellanic penguins in northern Patagonia.

## 2 Study Area

The Islote Lobos National Park (ILNP; 41°26′ S, 65°26′ W) is located along the Atlantic coast of San Matías Gulf, Río Negro Province, Argentina, approximately 50 km east of the city of Sierra Grande. The protected area encompasses a group of six small granitic and sedimentary islets—Lobos, La Pastosa, Redondo, Ortiz Norte, Ortiz Sur and Isla de los Pájaros—situated a short distance offshore (Figure 1). La Pastosa is the largest islet in the group, whereas Islote Lobos represents the northernmost unit.

**FIGURE 1:**
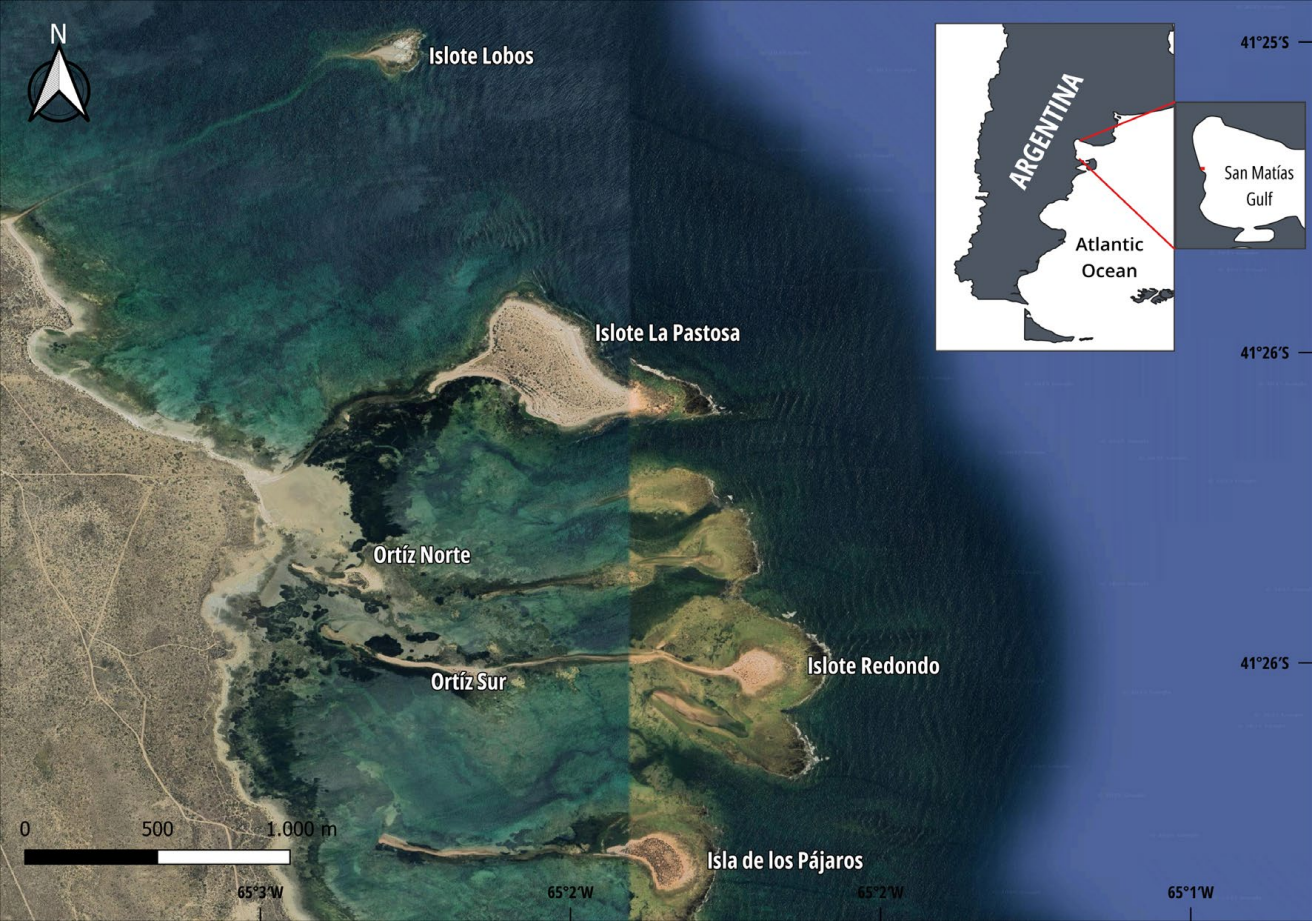
Islote Lobos National Park (ILNP) showing the distribution of the six islets (Lobos, La Pastosa, Redondo, Ortiz Norte, Ortiz Sur and Isla de los Pájaros). Satellite imagery: Google Earth.

During low tide, the islet complex becomes temporarily connected to the mainland through tombolos and sediment bars, generally for periods of 3–4 h two times each day, exposing a broad intertidal zone composed of sandy areas, rocky patches, and tidal pools. The semidiurnal tidal regime (6–9 m amplitude) generates variable accessibility to the islets and enables occasional access by terrestrial mammals, including pumas, foxes, and wild boar ( *Sus scrofa* ), during low‐tide periods.

The islets differ in substrate composition and vegetation cover. Lobos is entirely rocky and lacks vegetation, whereas the remaining islets consist mainly of recent sedimentary deposits (sand, shell fragments, and guano) supporting dense shrub vegetation, mainly composed of jume *Suaeda divaricata*, zampa *Atriplex lampa* , jarilla *Larrea* spp. and yaoyín *Lycium chilense*. Three islets—La Pastosa, Redondo, and Isla de los Pájaros—host breeding colonies of Magellanic penguins, representing the entire breeding population of the species within the park. In 2024, population surveys conducted by the authors estimated approximately 11,800 breeding pairs across the three colonies (Hombre et al., in prep.). Monitoring campaigns continued during the 2025 breeding season, during which the predation events described here were recorded.

## 3 Field Observations

Observations were obtained incidentally during long‐term monitoring campaigns of Magellanic penguin breeding colonies at ILNP. Population monitoring focused on colony abundance and reproductive success has been conducted annually during four consecutive breeding seasons prior to and including the events reported here. Field teams accessed the islets on foot during low‐tide windows and surveyed nesting areas between late October and mid‐January during both breeding seasons considered in this study (2024–2025 and 2025–2026).

During each visit, carcasses were inspected in situ to assess external injuries and evidence of consumption. When relevant, GPS coordinates and photographs were recorded, and carcasses were marked to avoid repeated counts during subsequent surveys. No carcasses were collected and no necropsies were performed. Due to the park's internal regulations, field observations relied exclusively on non‐invasive procedures, and no wildlife handling or manipulation.

### 3.1 2024–2025 Season

On 30 October 2024, during a field campaign at Isla de los Pájaros, four adult Magellanic penguins were found dead within the breeding colony. None of the carcasses showed signs of consumption, and all exhibited cervical bite injuries characteristic of felid attacks (Figure 2A). No fresh tracks or other direct evidence of the predator were detected during the survey.

**FIGURE 2:**
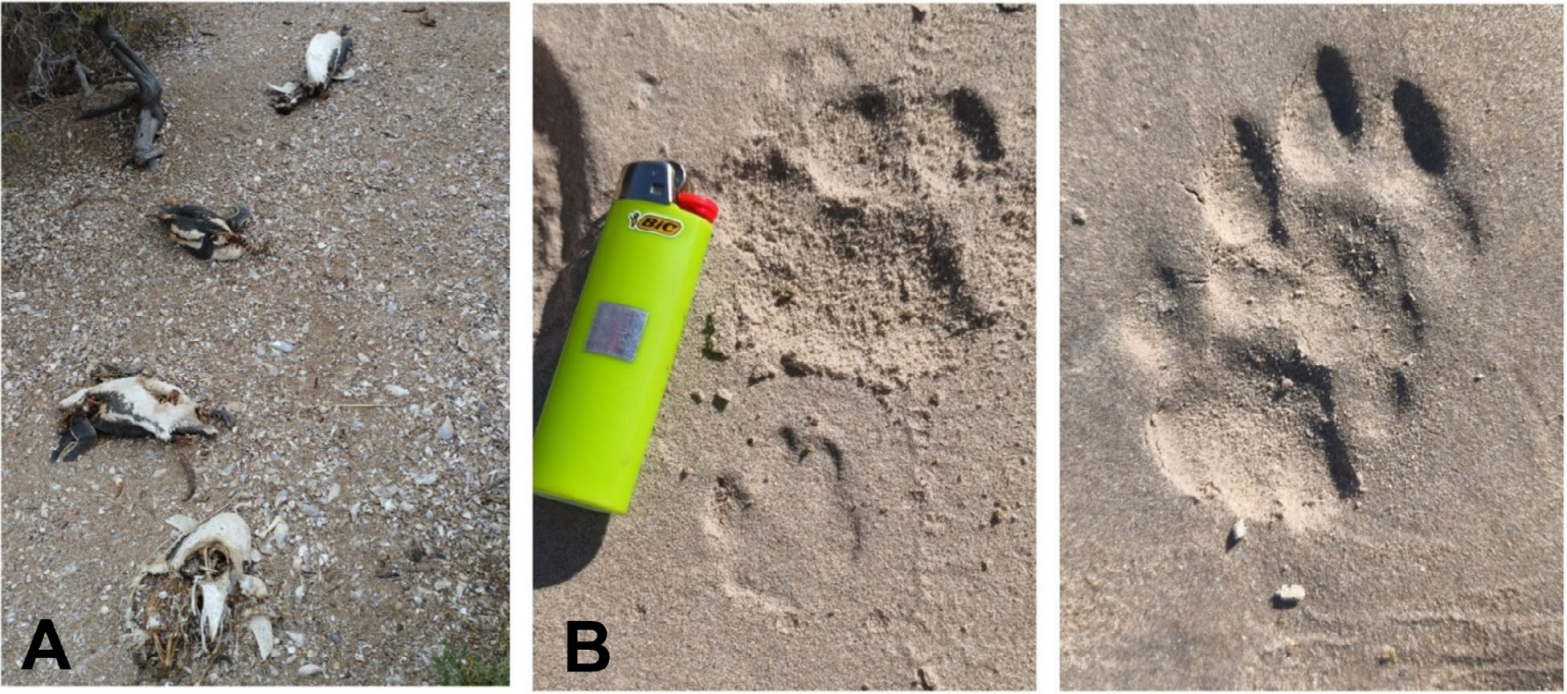
Evidence associated with puma predation on Magellanic penguins at Islote Lobos National Park. (A) Four adult Magellanic penguin carcasses showing cervical injuries and no signs of consumption, recorded at Isla de los Pájaros during the 2024–2025 breeding season. (B) Fresh tracks of *Puma concolor* recorded in the intertidal zone during the 2025–2026 breeding season. A lighter (8 cm long) is included for scale; tracks of a gray fox ( *Lycalopex griseus* ), a common resident of the islets, are also visible.

According to park ranger records and field observations from that period, pumas had been inferred to be present in the area based on indirect signs, including tracks and resting marks, although no direct sightings were confirmed. Subsequent monitoring visits conducted throughout the remainder of the breeding season (November–December 2024) did not reveal additional predation events or new carcasses, indicating that the observed event was temporally restricted.

### 3.2 2025–2026 Season

The first event, recorded on 20 October at La Pastosa islet, involved the detection of fresh puma tracks imprinted in wet sand along the intertidal access route to the colony and oriented inland. The tracks exhibited sharply defined edges and recently displaced sand, with no signs of tidal overwash or erosion, indicating that they had been produced during the most recent low‐tide period, shortly before the survey (Figure 2B). In addition, park staff and researchers had accessed the islet 2 days earlier (18 October) and reported no evidence of penguin mortality at that time, further supporting the recent occurrence of the event.

In the southwestern sector of the colony, approximately 185 adult penguins were found dead, scattered among nests and vegetation (Figure 3A).

**FIGURE 3:**
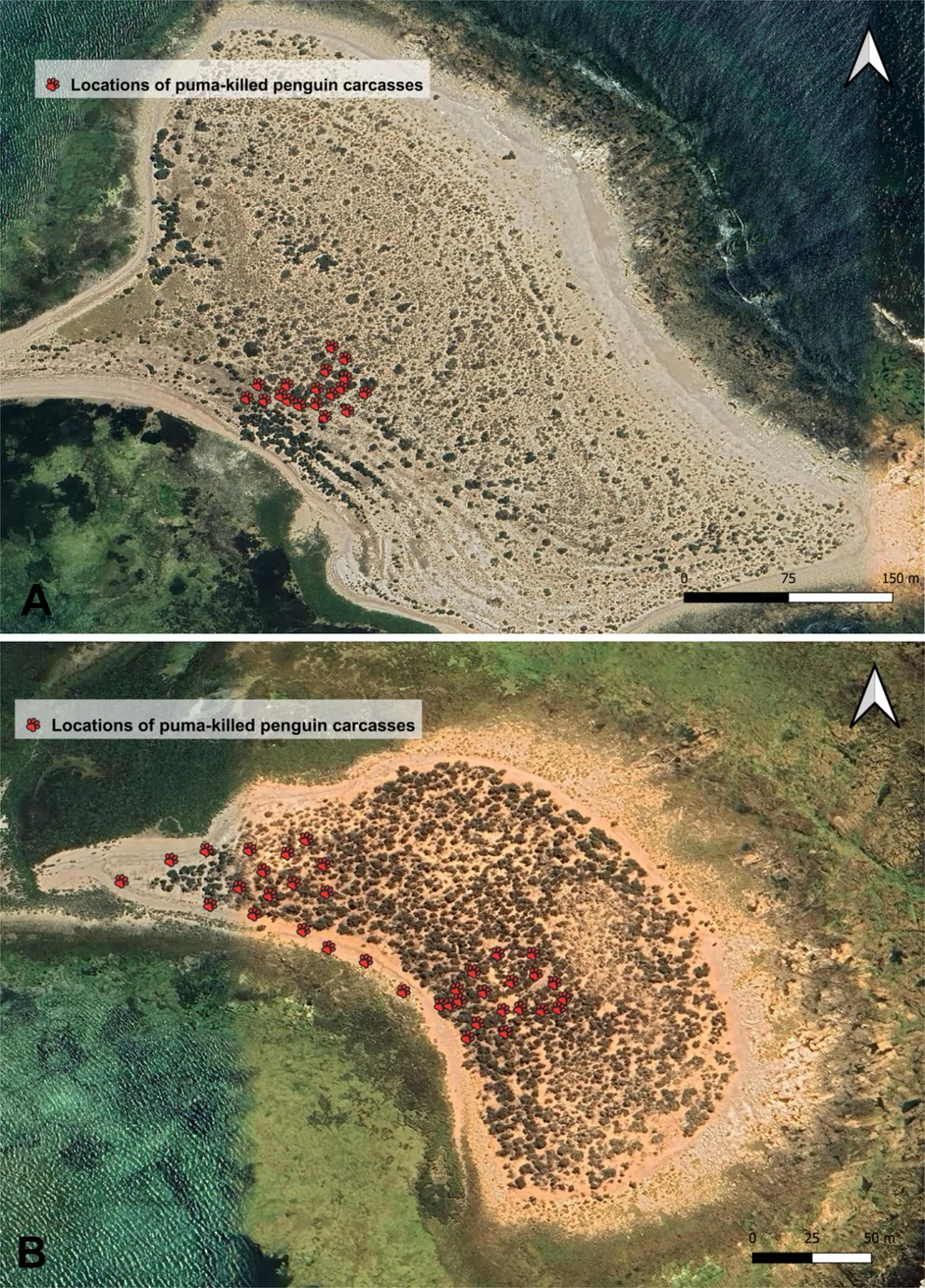
Spatial distribution of penguin carcasses killed by pumas during the 2025 breeding season at (A) La Pastosa islet and (B) Isla de los Pájaros. Red symbols indicate carcass locations. Satellite imagery: Google Earth.

The carcasses exhibited deep bite marks and cervical lacerations, with some individuals partially or completely decapitated, whereas the remainder of the body remained intact, showing fresh blood and no signs of consumption (Figure 4A–C). These injuries were consistent with predation by a large felid and coincided with the presence of fresh puma tracks recorded at the intertidal access point to the islet.

**FIGURE 4:**
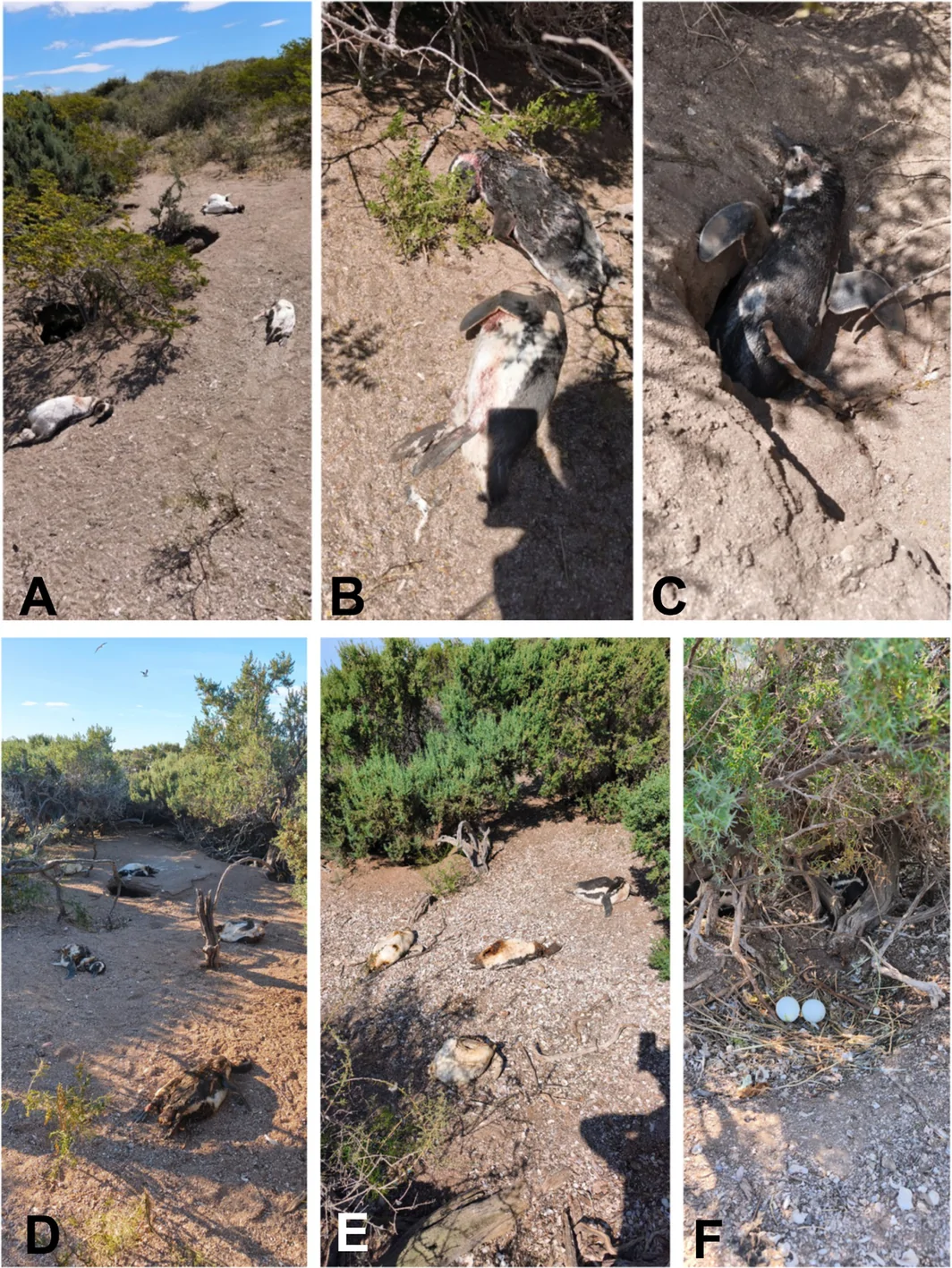
Magellanic penguins preyed upon by pumas and associated nest impacts during the 2025–2026 breeding season. (A–C) La Pastosa islet: preyed‐upon penguins recorded inside and outside nests. (D–F) Isla de los Pájaros: preyed‐upon penguins and nests with abandoned eggs.

The second event was recorded the following day, 21 October, on Isla de los Pájaros, where around 190 dead penguins were found, distributed from the shoreline into the interior of the colony (Figure 3B).

Carcasses showed more advanced decomposition relative to those at La Pastosa islet, suggesting that predation had occurred within a few days prior to the survey. In many areas, active nests contained abandoned eggs, and in some cases entire breeding pairs were found dead or a surviving adult remained next to its deceased mate.

The spatial distribution of carcasses and the absence of consumption closely resembled the pattern observed at La Pastosa islet, suggesting a comparable predation dynamic between both events (Figure 4D–F).

These observations derive from opportunistic field records obtained during penguin monitoring campaigns, rather than from targeted surveys of puma activity.

Additional predation events were subsequently documented by park rangers during late January and early February 2026.

On 22 January 2026, a new mortality event was recorded at La Pastosa islet, where numerous dead penguins exhibiting bite marks and cervical injuries consistent with felid attacks were detected (Figure 5A). Two additional surveys conducted between 24 and 28 January documented further mortality events within the same colony, resulting in a cumulative total of 420 dead Magellanic penguins, including 361 adults and 59 chicks (APN 2026a). During these surveys, fresh puma tracks and other signs of predator activity were repeatedly recorded (Figure 6), and carcasses were systematically marked to avoid repeated counts.

**FIGURE 5:**
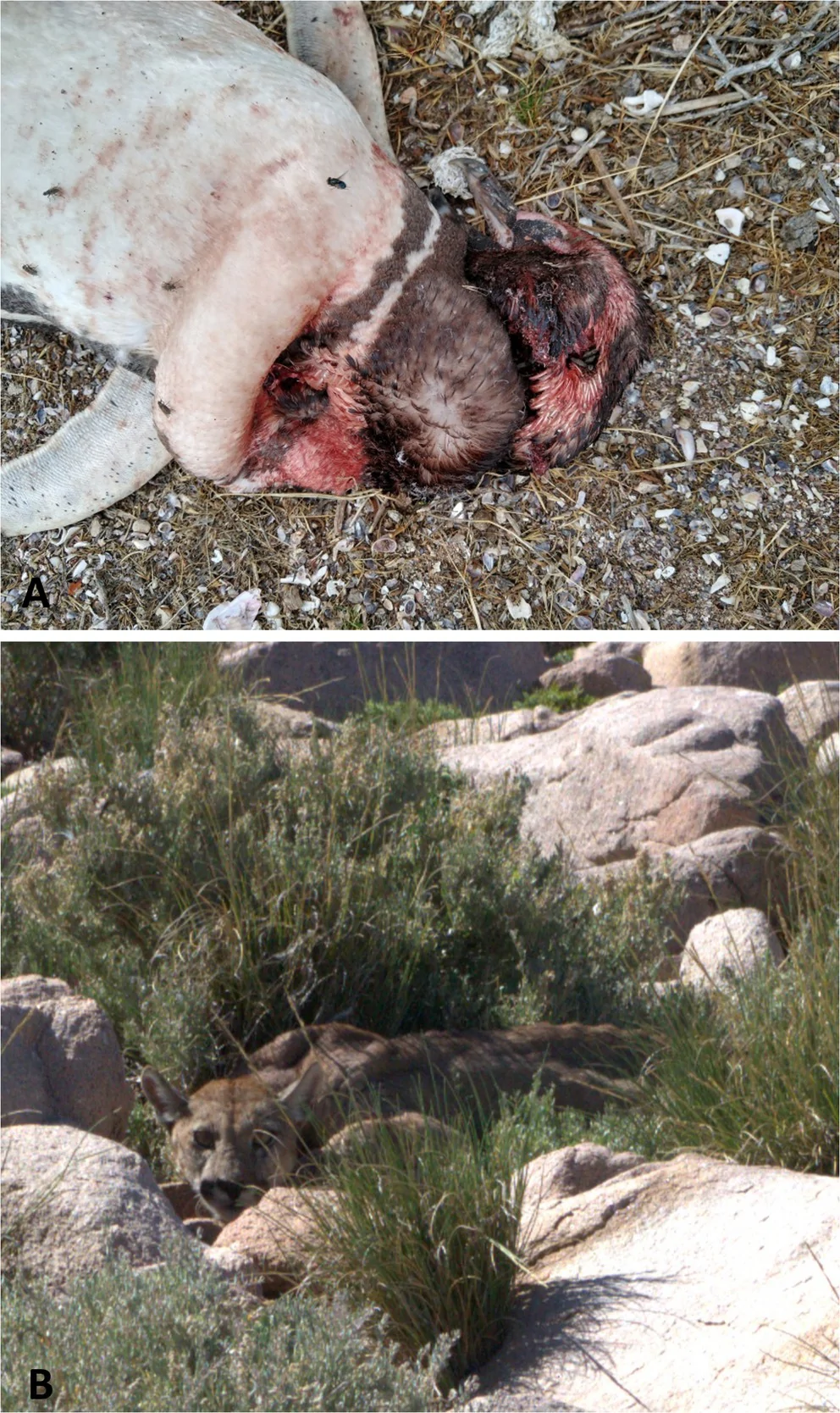
Evidence associated with puma predation during the 2025–2026 breeding season. (A) Cervical injuries and partial decapitation of an adult Magellanic penguin consistent with a felid attack. (B) Injured adult puma recorded within the penguin colony during the February 2026 surveys. Photo credit: Park Ranger Matías Maldonado.

**FIGURE 6:**
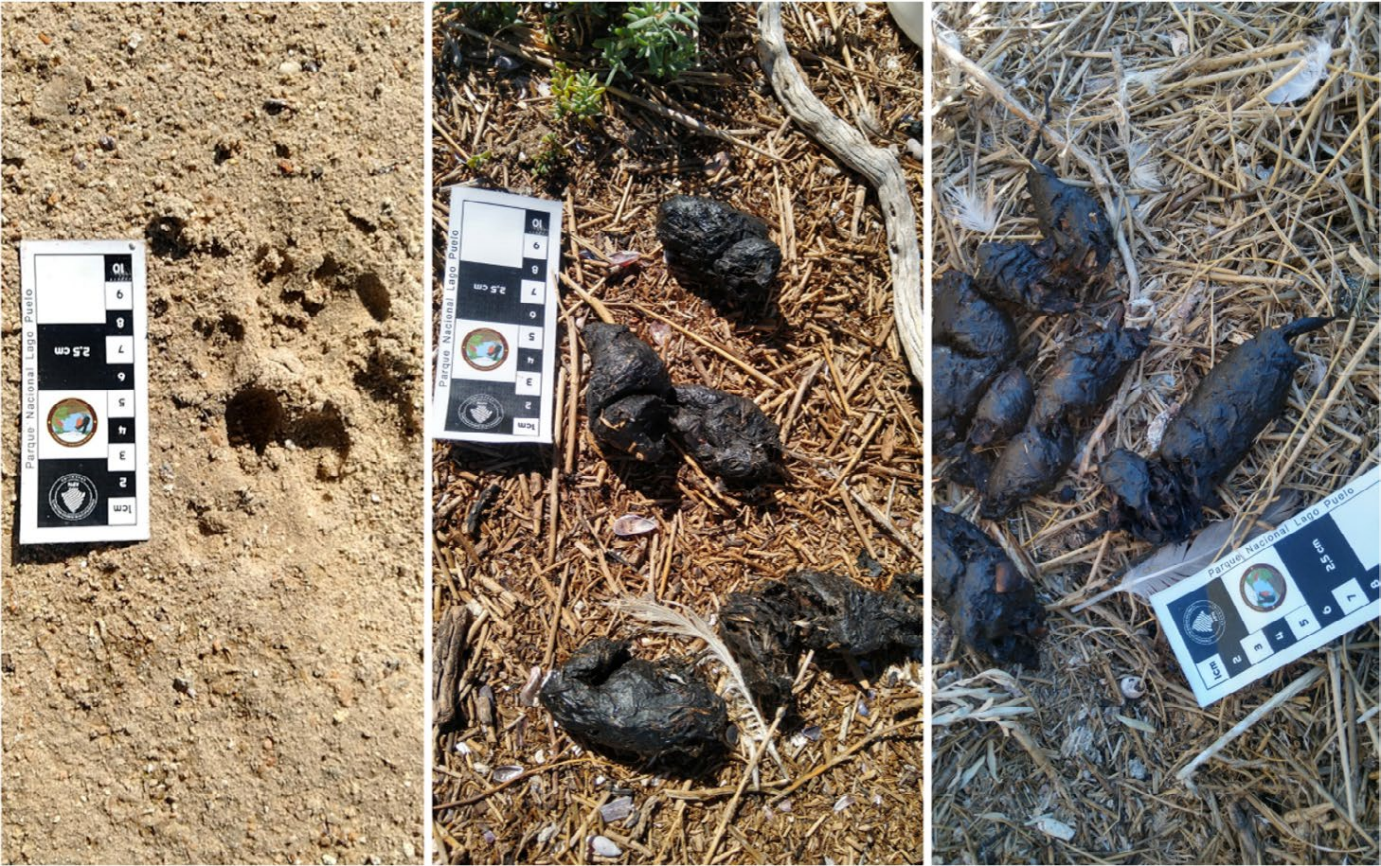
Signs of puma activity recorded during January 2026 surveys at La Pastosa islet. Left: fresh puma track in sandy substrate. Center and right: puma feces recorded within the penguin colony. Scale bars are shown in each panel (Photo credit: Park Ranger Matías Maldonado).

During one of the January surveys, park rangers directly observed an adult puma moving within the colony and resting inside penguin nesting areas. The individual showed evident signs of poor physical condition, including marked emaciation and an exposed fracture in the left hind limb. Given the physical condition of this animal and the magnitude and recurrence of the events, more than one individual may have been involved. Tracks of apparently different sizes were observed during the surveys; however, because track dimensions were not systematically measured across events, these observations cannot be used to reliably determine the number of pumas involved.

Additional mortality events were later documented at Isla de los Pájaros during surveys conducted on 5–6 February 2026. These surveys recorded at least 166 additional dead penguins (151 adults and 15 chicks), distributed across much of the islet perimeter and concentrated mainly near intertidal access sectors (APN 2026b). Puma feces were also recorded within the colony, and park rangers directly observed the same injured puma leaving the islet through the intertidal zone (Figure 5B).

Overall, at least 961 Magellanic penguins were recorded dead during the 2025–2026 breeding season across all documented predation events, including 887 adults and 74 chicks (Table 1). Considering the 2025 abundance estimate of 12,612 breeding pairs across the three colonies, equivalent to approximately 25,224 breeding adults, the estimated adult mortality represents a minimum direct mortality of 3.52% of the local breeding population. This estimate does not include indirect chick mortality associated with nest abandonment or orphaning following adult deaths and should therefore be considered conservative in demographic terms.

**TABLE 1:** Summary of Magellanic penguin mortality events recorded during the 2025–2026 breeding season at Islote Lobos National Park.

Date	Islet	Adults	Chicks	Total
20 Oct 2025	La Pastosa	185	0	185
21 Oct 2025	Isla de los Pájaros	190	0	190
22 Jan 2026	La Pastosa	262	41	303
24 Jan 2026	La Pastosa	61	17	78
28 Jan 2026	La Pastosa	38	1	39
5–6 Feb 2026	Isla de los Pájaros	151	15	166
Total		887	74	961

## 4 Discussion

The events documented at Islote Lobos National Park fulfill the behavioral characteristics commonly associated with surplus killing (SK), including high prey availability, temporally concentrated attacks, and little or no prey consumption (Kruuk 1972; Lucherini et al. 2018). However, beyond its behavioral interpretation, the recurrence of multiple high‐intensity mortality events within a single breeding season suggests that SK may also represent an important source of additive mortality for local penguin colonies. Furthermore, considering that these SK events had not been previously reported for ILNP, this new source of mortality appears as a factor to be taken into account in future demographic studies.

The spatial configuration of the ILNP islets may facilitate occasional access by terrestrial predators. During low tide, temporary connections between the mainland and the islets provide access routes for pumas to breeding colonies that are otherwise surrounded by water. Once within the colonies, the seasonal concentration of breeding penguins provides a highly aggregated and vulnerable prey resource.

The characteristics of the mortality events strongly point to pumas as the predators responsible for the attacks recorded at ILNP. Repeated detections of fresh tracks and feces attributable to pumas within the colonies, the direct observation of an adult individual on the islets during the January and February surveys, and the presence of cervical bite injuries and partial decapitation consistent with attacks by large felids all support this interpretation. In addition, while red foxes *Lycalopex culpaeus* have been recorded in several southern Patagonian penguin colonies such as Monte León, Cabo Dos Bahías, and Cabo Vírgenes, this species is not known to occur within the ILNP area. Although gray foxes *Lycalopex griseus* are regularly present on the islets, they are unlikely to produce high‐intensity mortality events involving large numbers of adult penguins. Taken together, these observations strongly support puma killing as the most plausible cause of the mortality events described here.

In northeastern Patagonia, pumas are generalist predators whose diet may include both native and introduced prey. A study conducted in northeastern Chubut found that guanaco ( *Lama guanicoe* ) was the most important prey item in the puma diet, while sheep represented a smaller contribution to consumed biomass (Fernández and Baldi 2014).

Similar interactions between pumas and Magellanic penguins have been documented in southern Patagonia, particularly at Monte León National Park, where recent studies have examined their effects on puma spatial ecology, penguin reproductive performance, and population dynamics (Serota et al. 2023, 2025; Lera et al. 2026; Connor et al. 2026; Dodino et al. 2026). The events recorded at ILNP extend the confirmed geographic range of puma‐penguin SK by more than 1000 km northwards and document this interaction under a distinct spatial configuration, involving breeding colonies located on offshore islets that become temporarily accessible to terrestrial predators during low tide. In addition, repeated high‐intensity mortality events occurred at two different breeding islets within a single reproductive season. Together, these observations broaden the geographic and ecological context in which puma‐penguin interactions are known to occur along the Patagonian coast.

Although the demographic consequences of these events remain uncertain, repeated mortality pulses affecting breeding adults could potentially influence reproductive success and local population trends if they recur over consecutive seasons. This possibility is particularly relevant because adult survival represents a critical demographic parameter in long‐lived seabirds such as penguins (Boersma et al. 2013). During the first reproductive survey of the 2025–2026 season, several previously marked nests within areas affected by puma predation were found empty and had to be replaced with other active nests for subsequent monitoring. Because the adults associated with these previously marked nests were not found dead at the nests, we could not determine whether the empty nests corresponded to breeding adults among the carcasses recorded elsewhere in the colony. Therefore, these breeding failures cannot be directly attributed to puma predation, although some of the adults found dead along the coast may have belonged to those nests. In Monte León National Park, Lera et al. (2026) demonstrated that puma predation alone would not necessarily drive colony extinction under favorable demographic conditions, but that additive mortality may contribute to population declines when combined with low reproductive success or reduced juvenile survival. Our observations suggest that similar processes could potentially occur in northern Patagonia if these killing events become recurrent, particularly considering that the breeding population at ILNP is substantially smaller than that of Monte León, which hosts approximately 44,000 breeding pairs (Hombre et al. 2026).

Ongoing monitoring conducted at ILNP during the last four breeding seasons recorded total breeding population estimates ranging between approximately 9800 and 12,600 breeding pairs during 2022–2025, while overall population growth remained stable (*λ* = 1.009; 95% CI = 0.921–1.105) (Hombre et al., in prep.). Importantly, during the four consecutive breeding seasons in which the authors monitored these colonies, no mortality events comparable in magnitude to those documented during the 2025–2026 breeding season had previously been recorded, with the exception of the isolated 2024 event involving four adult penguins described here. Within this context, the repeated mortality events documented here represent a substantial source of adult mortality relative to local colony size and highlight the importance of continued demographic monitoring.

The reasons for the marked increase in predation during the 2025–2026 season remain unknown. No systematic estimates of puma abundance are currently available for the ILNP area, and the number of individuals using the islets remains unknown. We have no data indicating whether this period was associated with reduced terrestrial prey availability, increased puma abundance or competition, or unusual access conditions to the islets. The tidal connection between the mainland and the colonies provides a recurrent opportunity for access, but similar access conditions were also present in previous breeding seasons without comparable mortality events. Therefore, the factors triggering the 2025–2026 events cannot currently be identified.

The repeated detection of fresh puma tracks at intertidal access sectors highlights the importance of tidal dynamics in facilitating access of terrestrial predators to marine bird colonies within coastal ecosystems.

The occurrence of these events may be related to the opportunistic behavior of pumas when encountering breeding colonies, where large numbers of highly vulnerable individuals are concentrated within a restricted area during a predictable period of time. Although Magellanic penguins do not appear to constitute a regular prey item for this species, these conditions may favor the occurrence of events such as those documented here. Events similar to those described here have also been documented at Monte León, Monte Entrance, and Punta Tombo colonies (Zanón Martínez et al. 2012; Serota et al. 2023; Lera et al. 2026). Together, these observations suggest that predator incursions into seabird colonies may represent a broader ecological phenomenon along the Patagonian coast.

Previous studies have suggested that reductions in livestock production and decreased predator persecution may facilitate the recolonization of coastal environments by pumas and other native carnivores (Walker and Novaro 2009; Ciancio et al. 2024). Protected‐area status could also potentially favor the occurrence of native carnivores indirectly through reduced persecution and lower levels of livestock‐related disturbance. However, no information is currently available to determine whether the recent establishment of ILNP has influenced puma occurrence in the area. Therefore, the presence of pumas within the park cannot be attributed to protected‐area status based on the available evidence. Within this framework, the events recorded at ILNP are best interpreted as part of the natural ecological dynamics of coastal ecosystems. Rather than supporting management actions directed at native species, the documentation of such events is essential to improve our understanding of how large carnivores exploit temporally and spatially concentrated resources, and how these interactions may shape the functional dynamics of seabird colonies along the Patagonian coast.

In summary, beyond the value of the information reported in this work for demographic studies of the Magellanic penguin, one aspect to highlight is that the occurrence of SK may be favored by ecological conditions that provide predators with access to large numbers of vulnerable and highly aggregated prey. SK by pumas has been associated with situations in which prey are abundant and easily accessible, potentially allowing repeated attacks beyond immediate energetic requirements (Lucherini et al. 2018). Such behavior does not necessarily imply an immediate energetic gain or a specific adaptive benefit, as capture and killing responses may persist when vulnerable prey remain readily available even after satiation (Kruuk 1972). At ILNP, the seasonal concentration of breeding penguins within spatially restricted colonies, together with temporary access from the mainland during low tide, may create such conditions. The events documented here therefore highlight how episodic access by terrestrial predators to highly aggregated marine prey can generate substantial mortality pulses, reinforcing the importance of considering these interactions in demographic monitoring of coastal seabird colonies.

## Author Contributions

**Jesica D. Hombre:** conceptualization (lead), data curation (equal), investigation (equal), visualization (lead), writing – original draft (lead). **Magdalena Arias:** investigation (equal), writing – review and editing (equal). **Esteban Tazzioli:** investigation (equal), writing – review and editing (equal). **Raúl González:** resources (equal), supervision (equal), writing – review and editing (equal). **Enrique Crespo:** supervision (equal), writing – review and editing (equal).

## Funding

This study was supported by the National Scientific and Technical Research Council (CONICET; Project PIP No. 11220200101942) and the National Parks Administration (APN).

## Conflicts of Interest

The authors declare no conflicts of interest.

## Supporting information


**Data S1:** ece374585‐sup‐0001‐DataS1.xlsx.

## Data Availability

All data supporting the findings of this study are included within the manuscript.

## References

[ece374585-bib-0001] Administración de Parques Nacionales (APN) . 2026a. “Informe de Relevamiento de Eventos de Caza de Pingüinos de Magallanes ( *Spheniscus magellanicus* ) por Puma ( *Puma concolor* ) en los Islotes del Parque Nacional Islote Lobos.” Informe técnico IF‐2026‐11460947‐APN‐PNIL#APNAC. Río Negro, Argentina.

[ece374585-bib-0002] Administración de Parques Nacionales (APN) . 2026b. “Informe de Relevamiento de Eventos de Caza de Pingüinos de Magallanes ( *Spheniscus magellanicus* ) por Puma ( *Puma concolor* ) en el Islote Pájaros del Parque Nacional Islote Lobos.” Informe Técnico IF‐2026‐14097514‐APN‐PNIL#APNAC. Río Negro, Argentina.

[ece374585-bib-0004] Boersma, P. D. , E. Frere , O. Kane , et al. 2013. Magellanic Penguin ( *Spheniscus magellanicus* ). University of Washington Press.

[ece374585-bib-0005] Ciancio, J. E. , A. Travaini , and E. Frere . 2024. “Consequences of Terrestrial Top Predator Control by Patagonian Sheep Farmers for Coastal Marine Food Webs.” Journal for Nature Conservation 82: 126747. 10.1016/j.jnc.2024.126747.

[ece374585-bib-0006] Connor, T. , P. A. Alarcón , M. Serota , et al. 2026. “Exploring the Potential Population Impacts of Puma Predation on a Mainland Magellanic Penguin Colony Through an Interactive Tool.” Ecological Informatics 96: 103793.

[ece374585-bib-0007] Dodino, S. , I. Barbe , G. Roskos , et al. 2026. “Disentangling Drivers of Magellanic Penguin Demography: Climate Variability and Terrestrial Predation at Southern Patagonia.” Frontiers in Marine Science 13: 1872508.

[ece374585-bib-0008] Fernández, C. S. , and R. Baldi . 2014. “Hábitos Alimentarios del Puma ( *Puma concolor* ) e Incidencia de la Depredación en la Mortandad de Guanacos ( *Lama guanicoe* ) en el Noreste de la Patagonia.” Mastozoología Neotropical 21, no. 2: 331–338.

[ece374585-bib-0025] García‐Borboroglu, P. , L. M. Pozzi , A. M. Parma , P. Dell’Arciprete , and P. Yorio . 2022. “Population Distribution Shifts of Magellanic Penguins in Northern Patagonia, Argentina: Implications for Conservation and Management Strategies.” Ocean and Coastal Management 222: 106156. 10.1016/j.ocecoaman.2022.106156.

[ece374585-bib-0009] Hombre, J. D. , M. Arias , M. F. Carrasco , R. González , and E. A. Crespo . 2026. “Status and Global Population Trend of the Magellanic Penguin *Spheniscus magellanicus* Along the Argentine Coast.” Scientific Reports 16: 3743. 10.1038/s41598-025-33756-3. PMC12852185 41519960

[ece374585-bib-0010] Jones, J. 2001. “Habitat Selection Studies in Avian Ecology: A Critical Review.” Auk 118, no. 2: 557–562. 10.1642/0004-8038(2001)118[0557:HSSIAE]2.0.CO;2.

[ece374585-bib-0011] Kruuk, H. 1972. “Surplus Killing by Carnivores.” Journal of Zoology 166: 233–244.

[ece374585-bib-0012] Lera, M. , E. Frere , J. Marino , A. Morgenthaler , S. Lai , and A. Millones . 2026. “Shifting Predator–Prey Dynamics at the Land–Sea Interface: The Case of Magellanic Penguins and Pumas.” Journal for Nature Conservation 91: 127208. 10.1016/j.jnc.2025.127208.

[ece374585-bib-0013] Linnell, J. D. C. , J. Odden , M. E. Smith , R. Aanes , and J. E. Swenson . 1999. “Large Carnivores That Kill Livestock: Do Problem Individuals Really Exist?” Wildlife Society Bulletin 27, no. 3: 698–705.

[ece374585-bib-0014] Lucherini, M. , M. M. Guerisoli , and E. M. Luengos Vidal . 2018. “Surplus Killing by Pumas ( *Puma concolor* ): Rumours and Facts.” Mammal Review 48, no. 4: 277–283. 10.1111/mam.12131.

[ece374585-bib-0015] Murphy, T. , and D. W. Macdonald . 2010. “Pumas and People: Lessons in the Landscape of Tolerance From a Widely Distributed Felid.” In Biology and Conservation of Wild Felids, edited by D. W. Macdonald and A. Loveridge , 431–445. Oxford University Press.

[ece374585-bib-0017] Polis, G. , and S. Hurd . 1996. “Allochtonous Input Across Habitats, Subsidized Consumers, and Apparent Trophic Cascades: Examples From the Ocean‐Land Interface.” In Food Webs: Integration of Patterns and Dynamics, edited by G. Polis and K. Winemiller , 275–285. Chapman & Hall.

[ece374585-bib-0020] Serota, M. W. , P. A. E. Alarcón , E. Donadio , and A. D. Middleton . 2023. “Puma Predation on Magellanic Penguins ( *Spheniscus magellanicus* ): An Unexpected Terrestrial–Marine Linkage in Patagonia.” Food Webs 36: e00290. 10.1016/j.fooweb.2023.e00290.

[ece374585-bib-0021] Serota, M. W. , T. Connor , W. Xu , P. A. Alarcón , E. Donadio , and A. D. Middleton . 2025. “A Marine Subsidy Reshapes the Ecology of a Large Terrestrial Carnivore.” Proceedings. Biological Sciences 292, no. 2061: 20252172. 10.1098/rspb.2025.2172 41538027

[ece374585-bib-0024] Walker, S. , and A. Novaro . 2009. “The World's Southernmost Pumas in Patagonia and the Southern Andes.” In Cougar: Ecology and Conservation, edited by M. Hornocker , S. Negri , and A. Rabinowitz , 91–99. University of Chicago Press.

[ece374585-bib-0023] Zanón Martínez, J. I. , A. Travaini , S. C. Zapata , D. E. Procopio , and M. Á. Santillán . 2012. “The Ecological Role of Native and Introduced Species in the Diet of the Puma ( *Puma concolor* ) in Southern Patagonia.” Oryx 46, no. 1: 106–111. 10.1017/S0030605310001821.

